# Outcomes of Paclitaxel-Coated Balloon Angioplasty vs. Drug-Eluting Stents in the Management of Acute and Chronic Coronary Syndromes in All Vessel Sizes: A Propensity-Matched Study (The OUTDES Study)

**DOI:** 10.3390/jcdd13070327

**Published:** 2026-07-13

**Authors:** Upul Wickramarachchi, Natasha Corballis, Timothy Gilbert, Alisdair Ryding, Toomas Sarev, Trevor Wistow, Marcus Flather, Simon Eccleshall

**Affiliations:** 1 Norfolk and Norwich University Hospital, Norwich NR4 7UY, UK; natasha.corballis@nnuh.nhs.uk (N.C.); doctor@kardiostar.com (T.S.); trevor.wistow@nnuh.nhs.uk (T.W.); marcus.flather@nnuh.nhs.uk (M.F.); simon.eccleshall@nnuh.nhs.uk (S.E.); 2 Norwich Medical School, University of East Anglia, Norwich NR4 7TJ, UK

**Keywords:** acute coronary syndrome, Non-ST elevation myocardial infarction, ST elevation myocardial infarction, stable angina, drug-eluting balloon/drug-coated balloon (DCB), drug-eluting stent, de novo coronary artery disease

## Abstract

Percutaneous coronary intervention (PCI) using drug-coated balloons (DCBs) may provide outcomes comparable to drug-eluting stents (DESs) due to the absence of a permanent implant and improved coronary artery remodelling. This study compared clinical outcomes of DCB-only angioplasty with DESs in a real-world setting. All patients undergoing PCI with DCBs or DESs for de novo disease were included in a propensity score-matched analysis using prospective and retrospective collected data from a single centre. The primary outcome was target lesion revascularisation (TLR) at 12 months. The secondary outcomes were major adverse cardiac events (MACEs) defined as a composite of all-cause death, myocardial infarction, or TLR at 12 months. Propensity matching produced 904 DCB lesions (719 patients) matched to 1424 DES lesions (1271 patients). The DCB group had smaller coronary arteries, shorter treated segments, and more bifurcation lesions. The mean age was 65 years, 22% of patients had prior MI, 16% had diabetes, and 58% had acute coronary syndromes. The rate of TLR at 12 months was as follows: 2.3% with DCBs; 2.5% with DESs (OR 0.86, *p* = 0.726, 95% CI 0.37–2.02). MACE was 8.2% with DCBs and 7.3% for DESs (OR 1.04, 95% CI 0.73–1.47). Results suggest comparable outcomes in patients who received paclitaxel DCBs compared to DESs without excess MACE, highlighting the need for randomised controlled trials.

## 1. Introduction

Bare metal stents (BMSs) were developed to address acute vessel closure and severe recoil after balloon-only coronary angioplasty [1]. Subsequently, drug-eluting stents (DESs) were developed in the late 1990s to reduce restenosis rates seen after BMS implantation and have become the main stay of percutaneous coronary intervention (PCI) [2,3]. Drug-coated balloons (DCBs) are semi-compliant balloons covered with a chemotherapeutic drug, usually paclitaxel or sirolimus, which is delivered into the vessel wall when the balloon is inflated in a coronary artery. DCBs may provide favourable vessel wall remodelling [4,5,6] due to the absence of a permanent metal implant. There are randomised trials and meta-analyses supporting its use in small vessel coronary disease [7,8,9,10]. Reduced length of dual antiplatelet treatment, absence of under expansion, reduced late stent malapposition, and restoration of normal vessel contour and curvatures are possible advantages of DCB angioplasty [11,12,13]. We sought to investigate the clinical outcomes of DCBs compared to DESs in a “real world” cohort of patients using a propensity-matched design.

## 2. Materials and Methods

### 2.1. Study Design and Patient Selection

This is a single-centre, retrospective, propensity-matched study carried out using prospectively collected data. Patients who received DCB-only angioplasty or second-generation or newer DESs (zotarolimus or everolimus eluting) for de novo coronary artery disease from 2009 to 2015 at our high-volume PCI centre were identified through the hospital database. Approvals from the UK National Research Ethics Committee, Health Research Authority, Confidentiality Advisory Group, and Healthcare Quality Improvement Partnership, UK, were obtained prior to commencement. Based on these approvals, individual anonymised patient data were permitted to be used in this report without the requirement for patient-level consent. As this was a retrospective registry, registration with clinical trial website was not considered necessary.

### 2.2. Definitions of Clinical Outcomes

The primary outcome was target lesion revascularisation (TLR), and secondary outcomes were a composite of major adverse cardiovascular events (MACEs) consisting of all-cause death, myocardial infarction (MI), or target lesion revascularisation (TLR) at 12 months, in keeping with the ARC-2 recommendation for device outcome reporting, which was updated in 2018 [14]. Additional secondary outcomes were individual components of MACE and target lesion thrombosis. TLR was defined as repeat PCI or bypass surgery of the target lesion for restenosis or other complication of the target lesion. Target lesion is defined as the treated segment from 5 mm proximal and 5 mm distal to the treated lesion (by visual assessment). All-cause mortality was defined as death from any cause. MI was a hospital diagnosis based on elevated troponin and associated features consistent with the Third Universal Definition and the Myocardial Ischaemia National Audit Programme, UK (MINAP). Additional definitions for target vessel revascularisation, acute vessel closure, and stent thrombosis are provided in Appendix A.

### 2.3. Percutaneous Coronary Intervention Protocols

The procedural aspects of PCI were at the discretion of the operator based on clinical judgement. The DCB angioplasty protocol was based on the German consensus guidelines [15]. The target lesion was pre-dilated with an appropriately sized balloon (balloon: artery ratio of 0.8–1:1), followed by deployment of the DCB if lesion preparation was successful with less than 30% recoil and not more than NHLBI type B coronary dissection. DES implantation was carried out using standard practice of pre-dilatation, deployment of stent, and post-dilatation at the discretion of the operator. Unfractionated heparin (70–100 IU/kg) was used during the procedure aiming for an activated clotting time (ACT) of 250 s. Adjunct devices, such as cutting or scoring balloons and rotational atherectomy, were used at the discretion of the operator. Aspirin and clopidogrel were used as dual antiplatelet therapy in stable coronary artery disease in both arms (1 year for DES and 1 month for DCB) and for 1 year for all patients with ACS. From 2013 onwards, ticagrelor has been used for ACS, and from 2014 onwards, the majority of ACS patients received aspirin and ticagrelor in both arms. 

### 2.4. Data Acquisition and Analysis

**Figure 1:**
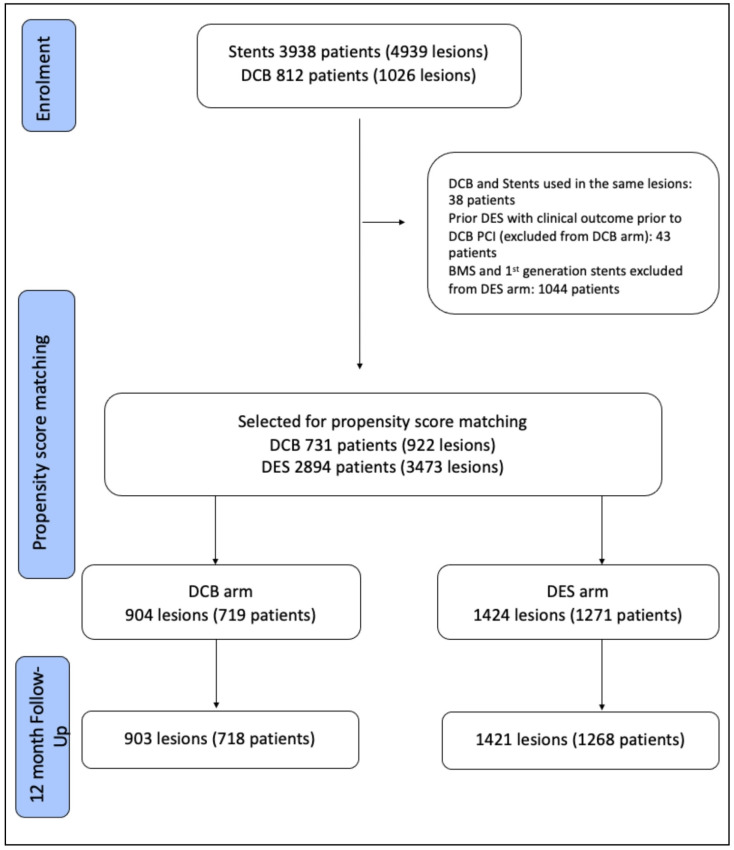
Patient flow diagram.

Demographics and procedural and lesion characteristics were identified from a prospectively completed hospital database. All patients who received either DES or DCB treatment during the above-mentioned period were included, and the exclusion criteria applied to compare the DES-only versus DCB-only strategies are shown in Figure 1.

Clinical outcomes, including vital status, were obtained from the UK National Institute for Cardiac Outcomes Research (NICOR) and NHS Digital. Data on all PCI procedures, CABG, and MIs in the UK are sent to NICOR as part of a UK national program of data collection. We obtained information on non-fatal events through the NICOR database and cross checked these with information available from records in our own centre. Local clinical records were then used to supplement data collection. If any discrepancies were found, they were evaluated by at least two members of the research team to determine validity and confirm or reject the event. 

### 2.5. Statistical Analysis

Propensity score matching was carried out using nearest neighbour matching within caliper algorithm to match each lesion in the DCB group with one or more (up to 3) lesions from the DES group [16]. The caliper threshold was 0.25 × SD and matching with replacement was carried out. A logistic regression model with the binary group indicator (DCB = 1, DES = 0) as the dependent variable was used to calculate propensity scores. The predictor variables for the propensity score model were: age, gender, diabetes, hypertension, previous MI, previous PCI, previous coronary artery bypass graft (CABG), primary PCI, indication (ACS/AMI/Stable), cardiogenic shock, out of hospital cardiac arrest (OOHCA), vessel treated, device diameter mean (SD), length of treated segment, bifurcation lesion, heavy calcification, diffuse disease, severe tortuosity, and presence of thrombus. All the outcome variables were coded as binary (0 = no event, 1 = event), and a logistic regression or mixed effects logistic regression (as appropriate) at a lesion level was used for the outcome analysis. All analyses were carried out using Stata (StataCorp. 2015. Stata Statistical Software: Release 14. College Station, TX, USA: StataCorp LP.). Exploratory analyses using conventional statistical comparisons, descriptive odds ratios, and 95% confidence intervals with *p*-values have also been provided where considered helpful. A lesion-level analysis was conducted for the primary endpoint of TLR. The composite endpoint of MACE was analysed at a patient level to avoid double counting of events.

## 3. Results

**Figure A1:**
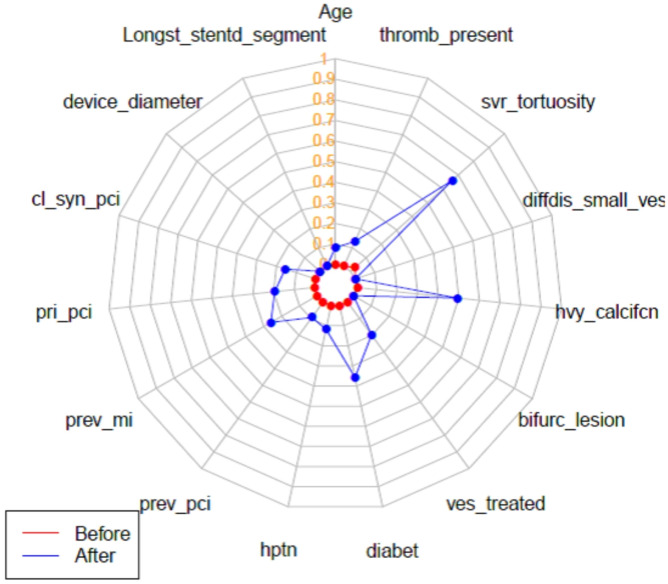
Distribution of *p*-values (in orange) of the covariates before and after propensity score matching. Prev—previous, MI—myocardial infarction, PCI—percutaneous coronary intervention, hptn—hypertension, diabet—diabetes mellitus, bifurc.—bifurcation, hvy—heavy, diffdis—diffuse disease, cl_syn—clinical syndrome, svr—severe, longst—longest, thromb—thrombus.

A total of 3938 patients (4939 de novo lesions) treated with DESs and 812 patients (1026 de novo lesions) treated with DCB angioplasty were identified from the hospital database from 2009 to 2015. In total, 38 patients who had a DCB, as well as a stent in the same lesion (±5 mm proximal or distal to index lesion), were excluded from the DCB arm. Another 43 patients who had received a prior drug-eluting stent (DES) and had a clinical event prior to DCB angioplasty were excluded from the DCB arm. A total of 1044 patients who received bare metal stents (BMSs) or first-generation DESs were excluded from the DES arm. Finally, 2894 patients (3473 lesions) treated with second-generation or newer DESs and 731 patients (922 lesions) treated with DCB-only PCI were identified for propensity score matching. A consort-style diagram in Figure 1 describes the patient flow. Pre-propensity matching patient and lesion characteristics are described in Appendix A, Table A1 and Table A2. Appendix A, Figure A1, shows the distribution of *p*-values of the covariates before and after propensity matching.

Of the 922 DCB-treated lesions, 794 (86.1%) were treated with SeQuent^®^ Please (B Braun Melsungen AG, Melsungen, Germany), 127 (13.8%) with In.Pact Falcon (Medtronic, Inc., Santa Rosa, CA, USA), and one with a DIOR balloon (Eurocor Tech GmbH, In den Dauen 6a, D-53117 Bonn, Germany). All drug-coated balloons were paclitaxel-coated. Gender, previous CABG, cardiogenic shock, and out-of-hospital cardiac arrest were not used in the propensity score model, as these covariates did not differ between the groups before matching. The mean age across the two groups was 65.2 years, 24% of patients were female, prior MI occurred in 22% of cases, diabetes was present in 16% of cases, hypertension was present in 49% of cases, and ACS was present in 57% of cases (24% primary PCI). The length of the treated segment, device diameter, diffuse disease, and bifurcation lesion covariates were significantly different in the two arms even after propensity score matching, but all other covariates were well matched (Table 1). A total of 61% of DCB lesions were treated with a device diameter of ≥3 mm.

Clinical outcomes after propensity score matching are described in Table 2. The primary outcome, TLR, occurred in 2.3% with DCBs and 2.5% with DESs (OR 0.86, *p* = 0.73, 95% CI 0.37–2.02). MACE rates at 12 months were 8.2% in the DCB group and 7.3% in the DES group (odds ratio [OR] 1.04, 95% CI 0.73–1.47). All-cause mortality was 3.6% in the DCB group and 2.9% in DESs (OR 1.10, CI 0.65–1.86, *p* = 0.72) and MI 2.6% vs. 3.2% (OR 0.74, CI 0.42–1.31, *p* = 0.74) (Unmatched clinical outcomes at 12 months are described in Appendix A Table A3). There were 4 (0.4%) acute vessel closure/re interventions on the same day in the DCB group and 4 (0.3%) in the DES group (OR 1.58, CI 0.39–6.32, *p* = 0.52).

## 4. Discussion

Our study shows that clinical outcomes after PCI with DCB-only (paclitaxel) angioplasty in de novo coronary artery disease of all vessel sizes, including acute coronary syndromes and elective PCI, are comparable to that of second-generation or newer DESs. The DCB population includes 61% of patients with coronary arteries ≥3 mm diameter and with angiographically more complex coronary disease. Of particular importance is the low acute vessel closure rate in the DCB group, which is in line with modern DES data, despite this cohort including a learning curve for many operators in our centre.

We found that the proportion of primary PCI patients and lesions with thrombus were higher in the DES group pre-propensity matching. This may reflect a degree of operator preference to stent more patients in the acute occlusion setting early in their individual learning curves of DCB-only PCI. However, after propensity score matching, most covariates were comparable for the two cohorts, apart from length of the treated segment, device diameter, bifurcation status, and presence of diffuse disease. There appeared to be systematic differences between the two groups for these parameters which could not be completely corrected by propensity score matching and suggest a persisting higher lesion complexity in the DCB group. This may also reflect operator preference to use DCBs in those subgroups where a DCB-only strategy is attractive, in particular for smaller vessels, diffuse disease, and bifurcations.

The BELLO (Balloon Elution and Late Loss Optimization) trial randomised 182 patients with coronary lesions in vessels <2.8 mm to paclitaxel DCBs or DESs and showed that late luminal loss at 6 months was non-inferior in the DCB group, with MACE rates of 7.8% vs. 13.2% (NS), respectively [17]. The BASKET-SMALL randomised trial showed non inferiority for MACE (cardiac death, MI, TVR) in DCB-only angioplasty compared to DESs in coronary arteries of 2–3 mm diameter in 382 and 376 patients, respectively [8]. MACE rates at 12 months in our study are consistent with the MACE rates in BASKET-SMALL (7.5% DCBs vs. 7.3% DESs, respectively). Another study from China randomised 170 patients with coronary diameter >3 mm to paclitaxel DCBs or new-generation DESs and showed that late lumen loss was non-inferior in the DCB group at 9 months with MACE rates of 2.44% vs. 6.33% (NS), respectively [18]. The SCAAR registry showed no significant difference in target lesion restenosis (a new, clinically relevant >50% stenosis in a previously treated segment assessed visually or by a significant reduction in fractional flow reserve or instantaneous wave-free ratio) when DCB angioplasty was compared to second-generation DESs, in a propensity-matched cohort of 1197 lesions in each arm at a median follow-up of 901 days, rates of target lesion restenosis were 7.0% vs. 6.2% in the DCB and DES groups respectively, but there was significantly less target lesion thrombosis in the DCB group (0.2% vs. 1.1% respectively, adjusted HR 0.18; 95% CI 0.04–0.82). However, the SCAAR registry did not provide information on standard parameters like TLR, TVR or MI and included just 5.8% of lesions with diameter >3 mm treated with a DCB, indicating mainly a smaller vessel cohort, whereas our population had 61% of lesions treated with a DCB diameter of 3 mm or more [19]. Our study adds to this knowledge by analysing clinical outcomes after a DCB in a wide range of coronary artery diameters and clinical indications for PCI. The REC-CAGEFREE 1 trial randomised 2272 patients (de novo all vessel sizes) to DCBs (*n* = 1133) or DESs (*n* = 1139) and failed to show non-inferiority at two years follow-up. However, it is noteworthy that the Swide DCB (Shenqi Medical, Shanghai, China) used a spray-coating technique of paclitaxel and iopromide on the balloon, which may have affected its efficacy [20].

Certain lesion characteristics, such as a long segment of disease or diffuse disease, may benefit more from DCBs in contrast to a long DES, which is known to be associated with higher rates of TLR [21]. Patient characteristics such as high bleeding risk help determine those who would benefit from a DCB strategy, which is associated with lower bleeding rates, partly due to the shorter durations of dual antiplatelet therapy [11]. Mechanistic studies have helped to understand how DCBs may be associated with preservation of the vessel lumen, with OCT demonstrating healing of the dissection flap and plaque regression driving vessel enlargement [5]. This also suggests that DCB angioplasty may have a role where the true vessel size is unclear, such as chronic total occlusions or ST elevation. Bifurcation lesions are a particular subset of PCI procedures that carry a higher degree of complexity. The reliance on DESs to solve the anatomical and geometrical intricacies of a bifurcation add to this complexity. Our current data suggest that bifurcation DCB-only PCI is safe and feasible, which allows simplification of the procedure. Again, late lumen gain is an advantage and has been shown specifically in the ostia of side-branch lesions at follow-up angiography and OCT [22,23]. Another possible advantage with DCBs is a reduction in MI rate due to the absence of permanent metal/polymer (and associated stent-related factors such as fracture, overlap, bifurcation stents, under-expansion, and malapposition), reduced inflammation, and more complete endothelialisation.

## 5. Limitations

This is a single-center non-randomised observational study spanning from 2009 to 2015, reflecting early DCB experience. Even though the data was collected prospectively, the analysis is a retrospective one. Therefore, it is subject to inherent limitations such as residual confounding, selection bias, and limited generalisability. The temporal factors could have influenced the outcomes, as changes in DES platforms (data of which are not captured) and antiplatelet regimes took place during this period. Also, this is a clinical-outcomes study; therefore, no routine follow-up angiography/physiology or imaging studies have been performed. Finally, it is not a non-inferiority trial, and some outcomes carry wide confidence intervals; therefore, the our results should be treated as hypothesis-generating for future randomised trials.

## 6. Conclusions

This propensity-matched analysis generates the hypothesis that TLR and MACE rates at 12 months with DCB-only (paclitaxel coated) angioplasty are comparable to those of second-generation or newer DESs. The population studied included patients with acute and chronic coronary syndromes and different coronary artery sizes, with a high proportion (61%) of coronary arteries ≥3 mm. These findings support the conduct of multi-centre randomised trials to further investigate these findings.

## Figures and Tables

**Table 1:** Post-propensity score matching demographics and lesion characteristics.

Covariate	DCB 904 Lesions 719 Patients	DES 1424 Lesions 1271 Patients	*p*
Age (years)	65.8	64.9	0.99
Previous MI %	209 (23.1)	301 (21.1)	0.26
Previous PCI %	210 (23.2)	289 (20.3)	0.09
Hypertension %	463 (51.2)	682 (47.9)	0.12
Diabetes %	152 (16.8)	219 (15.4)	0.36
Indication Stable CAD % ACS %	399 (44.1) 505 (55.9)	586 (41.2) 838 (58.8)	0.16
Primary PCI %	209 (23.1)	363 (25.5)	0.20
Vessel treated LMS % LAD % Cx % RCA%	14 (1.5) 452 (50) 226 (25) 212 (23.5)	31 (2.20) 716 (50.3) 311 (21.8) 366 (25.7)	0.20
Length of the treated segment mm	23.7 (11.0)	25.6 (11.8)	<0.01
Device diameter mm	3.0 (0.6)	3.2 (0.5)	<0.01
Bifurcation lesion %	297 (32.9)	376 (26.4)	<0.01
Heavy calcification %	187 (20.7)	278 (19.5)	0.50
Diffuse disease %	340 (37.6)	445 (31.3)	<0.01
Severe tortuosity %	149 (16.5)	225 (15.8)	0.66
Thrombus present %	191(21.1)	339 (23.8)	0.13

DCB—drug-coated balloon, DES—drug-eluting stent, MI—myocardial infarction, PCI—percutaneous coronary intervention, CAD—coronary artery disease, ACS—acute coronary syndrome, LMS—left main stem, LAD—left anterior descending, RCA—right coronary artery.

**Table 2:** Clinical outcomes at 12 months post-propensity score matching, risk-adjusted.

	DCB	DES	OR	95% CI	*p*
Per lesion	903 lesions ^1^	1421 lesions ^2^			
TLR	21 (2.3%)	35(2.5%)	0.86	0.37–2.02	0.73
Definite lesion/stent thrombosis	0 (0%)	8 (0.6%)	0.09	0.01–1.60	0.10
Acute vessel closure/re-intervention on the same day	4 (0.4%)	4 (0.3%)	1.58	0.39–6.32	0.52
Per-patient outcomes	718 patients	1268 patients			
MACE (death, MI, TLR)	59 (8.2%)	93 (7.3%)	1.04	0.73–1.47	0.83
Death	26 (3.6%)	37 (2.9%)	1.10	0.65–1.86	0.72
MI	19 (2.6%)	41(3.2%)	0.74	0.42–1.31	0.74
TVR	34 (4.7%)	48 (3.8%)	1.12	0.71–1.78	0.63
Death/MI	43 (6.0%)	71 (5.6%)	0.99	0.66–1.48	0.96

^1^ One loss to follow-up. ^2^ Three losses to follow-up. DCB—drug-coated balloon, DES—drug-eluting stent, OR—odds ratio, CI—confidence interval, MACE—major adverse cardiac events, MI—myocardial infarction, TLR—target lesion revascularisation.

## Data Availability

Restrictions apply to the availability of these data. Data were obtained from the National Institute for Cardiovascular Outcomes Research (NICOR) UK and are available from the authors with the permission of NICOR UK.

## Abbreviations

The following abbreviations are used in this manuscript:

PCI	Percutaneous coronary intervention
DCB	Drug-coated balloon
DES	Drug-eluting stent
TLR	Target lesion revascularisation
MACE	Major adverse cardiac event
BMS	Bare metal stent
ACT	Activated clotting time
ACS	Acute coronary syndrome
CABG	Coronary artery bypass graft
MI	Myocardial infarction
OOHCA	Out-of-hospital cardiac arrest
CAD	Coronary artery disease
LMS	Left main stem
LAD	Left anterior descending
RCA	Right coronary artery
TVR	Target vessel revascularisation
OCT	Optical coherence tomography
CTO	Chronic total occlusion
STEMI	ST-elevation myocardial infarction

## Appendix A. Additional Definitions

Target vessel revascularisation (TVR): Any repeat percutaneous intervention or surgical bypass of any segment of the target vessel. The latter is defined as the entire major coronary vessel proximal and distal to the target lesion, including upstream and downstream branches and the target lesion itself.

Acute vessel closure: Repeat angioplasty (during the same hospital stay) for a complete or partial occlusion of the artery due to a dissection/thrombus.

Stent/treated lesion thrombosis: Categorised as acute (<1 day), subacute (1 to 30 days), and late (>30 days) and defined in parallel to the ARC guidelines on stent thrombosis (REF). Definite: Acute coronary syndrome and angiographic or pathological confirmation of thrombus in the previously treated lesion and 5 mm proximal or distal to it. Probable: Unexplained death ≤30 days or target vessel MI without angiographic information. Possible: Unexplained death >30 days after treatment.

Diffuse coronary artery disease: Lesion with a length of >20 mm with sequential narrowings of >50% severity on visual assessment. Heavy calcification: Visible heavy calcification in coronaries (visual assessment).

Tortuosity: Location in an angulated segment of >45 degrees or similar angulation of the proximal segment.

**Table A1:** Patient characteristics; pre-propensity score matching.

	DCB 731	%	DES 2894	%	*p*-Value
Age	65.8		64.2		0.999
Male gender	558	76.3	2213	76.5	0.939
Previous MI	159	21.8	479	16.6	0.001
Previous PCI	154	21.1	454	15.7	0.001
Previous CABG	33	4.5	104	3.6	0.243
Hypertension	371	50.8	1256	43.4	0.000
Diabetes	121	16.6	349	12.1	0.001
Indication Stable ACS/AMI	307 424	42.0 58.0	988 1906	34.1 65.9	0.000
Primary PCI	186	25.5	999	34.5	0.000
Cardiogenic shock	9	1.2	44	1.5	0.561
OOHCA	16	2.2	45	1.6	0.234

DCB—drug-coated balloon, DES—drug-eluting stent, MI—myocardial infarction, PCI—percutaneous coronary intervention, CABG—coronary artery bypass graft, ACS—acute coronary syndrome, AMI—acute myocardial infarction, OOHCA—out-of-hospital cardiac arrest.

**Table A2:** Lesion characteristics pre-propensity score matching.

	DCB 922 Lesions	%	DES 3473 Lesions	%	*p*-Value
Vessel treated LMS LAD Cx RCA	14 467 227 214	1.5 50.7 24.7 23.2	92 1600 694 1087	2.6 46.1 20.0 31.3	0.000
Length of treated segment	23.56 (10.9)		29.65 (16.4)		0.0000
Device diameter mean(SD)	2.9 (0.59)		3.4 (0.61)		0.0000
Bifurcation lesion	314	34.1	743	21.4	0.000
Heavy calcification	195	21.1	607	17.5	0.010
Diffuse disease	358	38.8	876	25.2	0.000
Severe tortuosity	158	17.1	495	14.3	0.029
Thrombus present	191	20.7	1020	29.4	0.000

DCB—drug-coated balloon, DES—drug-eluting stent, LMS—left main stem, LAD—left anterior descending, Cx—circumflex, RCA—right coronary artery, SD—standard deviation.

**Table A3:** Clinical outcomes at 12 months pre-propensity matching, unadjusted.

	DCB (922 Lesions, 731 Patients)	DES (3473 Lesions, 2894 Pts)	*p*-Value	OR	95% CI
TLR (per lesion)	21 (2.3%)	88 (2.5%)	0.66	0.90	0.55–1.45
MACE (death, MI, TLR)	60 (8.2%)	200 (6.9%)	0.23	1.20	0.89–1.63
Death	26 (3.6%)	71 (2.5%)	0.10	1.47	0.93–2.32
MI	20 (2.7%)	85 (2.9%)	0.77	0.93	0.57–1.52
TVR	34 (4.7%)	104 (3.6%)	0.18	1.31	0.88–1.95

DCB—drug-coated balloon, DES—drug-eluting stent, OR—odds ratio, CI—confidence interval, MACE—major adverse cardiac event, MI—myocardial infarction, TLR—target lesion revascularisation, TVR—target vessel revascularisation.

## References

[1] Fischman D.L., Leon M.B., Baim D.S., Schatz R.A., Savage M.P., Penn I., Detre K., Veltri L., Ricci D., Nobuyoshi M. (1994). A Randomized Comparison of Coronary-Stent Placement and Balloon Angioplasty in the Treatment of Coronary Artery Disease. N. Engl. J. Med..

[2] Serruys P.W., de Jaegere P., Kiemeneij F., Macaya C., Rutsch W., Heyndrickx G., Emanuelsson H., Marco J., Legrand V., Materne P. (1994). A Comparison of Balloon-Expandable-Stent Implantation with Balloon Angioplasty in Patients with Coronary Artery Disease. N. Engl. J. Med..

[3] Bønaa K.H., Mannsverk J., Wiseth R., Aaberge L., Myreng Y., Nygård O., Nilsen D.W., Kløw N.-E., Uchto M., Trovik T. (2016). Drug-Eluting or Bare-Metal Stents for Coronary Artery Disease. N. Engl. J. Med..

[4] Gershlick A.H. (2002). Treating Atherosclerosis: Local Drug Delivery from Laboratory Studies to Clinical Trials. Atherosclerosis.

[5] Kleber F.X., Schulz A., Waliszewski M., Hauschild T., Böhm M., Dietz U., Cremers B., Scheller B., Clever Y.P. (2014). Local Paclitaxel Induces Late Lumen Enlargement in Coronary Arteries after Balloon Angioplasty. Clin. Res. Cardiol..

[6] Cortese B., Silva Orrego P., Agostoni P., Buccheri D., Piraino D., Andolina G., Seregni R.G. (2015). Effect of Drug-Coated Balloons in Native Coronary Artery Disease Left with a Dissection. JACC Cardiovasc. Interv..

[7] Neumann F.-J., Sousa-Uva M., Ahlsson A., Alfonso F., Banning A.P., Benedetto U., Byrne R.A., Collet J.-P., Falk V., Head S.J. (2019). 2018 ESC/EACTS Guidelines on Myocardial Revascularization. EuroIntervention.

[8] Jeger R.V., Farah A., Ohlow M.-A., Mangner N., Möbius-Winkler S., Leibundgut G., Weilenmann D., Wöhrle J., Richter S., Schreiber M. (2018). Drug-Coated Balloons for Small Coronary Artery Disease (BASKET-SMALL 2): An Open-Label Randomised Non-Inferiority Trial. Lancet.

[9] Kleber F.X., Rittger H., Ludwig J., Schulz A., Mathey D.G., Boxberger M., Degenhardt R., Scheller B., Strasser R.H. (2016). Drug Eluting Balloons as Stand Alone Procedure for Coronary Bifurcational Lesions: Results of the Randomized Multicenter PEPCAD-BIF Trial. Clin. Res. Cardiol..

[10] Bruch L., Zadura M., Waliszewski M., Platonic Z., Eränen J., Scheller B., Götting B., Herberger D., Palmieri C., Sinicròpi G. (2016). Results from the International Drug Coated Balloon Registry for the Treatment of Bifurcations. Can a Bifurcation Be Treated without Stents?. J. Interv. Cardiol..

[11] Rissanen T.T., Uskela S., Eränen J., Mäntylä P., Olli A., Romppanen H., Siljander A., Pietilä M., Minkkinen M., Tervo J. (2019). Drug-Coated Balloon for Treatment of De-Novo Coronary Artery Lesions in Patients with High Bleeding Risk (DEBUT): A Single-Blind, Randomised, Non-Inferiority Trial. Lancet.

[12] Scheller B., Ohlow M.A., Ewen S., Kische S., Rudolph T.K., Clever Y.P., Wagner A., Richter S., El-Garhy M., Böhm M. (2020). Bare Metal or Drug-Eluting Stent versus Drug-Coated Balloon in Non-ST-Elevation Myocardial Infarction: The Randomised PEPCAD NSTEMI Trial. EuroIntervention.

[13] Vos N.S., Fagel N.D., Amoroso G., Herrman J.-P.R., Patterson M.S., Piers L.H., van der Schaaf R.J., Slagboom T., Vink M.A. (2019). Paclitaxel-Coated Balloon Angioplasty versus Drug-Eluting Stent in Acute Myocardial Infarction. JACC Cardiovasc. Interv..

[14] Garcia-Garcia H.M., McFadden E.P., Farb A., Mehran R., Stone G.W., Spertus J., Onuma Y., Morel M., van Es G.-A., Zuckerman B. (2018). Standardized End Point Definitions for Coronary Intervention Trials: The Academic Research Consortium-2 Consensus Document. Circulation.

[15] Thygesen K., Alpert J.S., Jaffe A.S., Simoons M.L., Chaitman B.R., White H.D., Thygesen K., Alpert J.S., White H.D., Jaffe A.S. (2012). Third Universal Definition of Myocardial Infarction. Eur. Heart J..

[16] Austin P.C. (2010). Statistical Criteria for Selecting the Optimal Number of Untreated Subjects Matched to Each Treated Subject When Using Many-To-One Matching on the Propensity Score. Am. J. Epidemiol..

[17] Latib A., Colombo A., Castriota F., Micari A., Cremonesi A., De Felice F., Marchese A., Tespili M., Presbitero P., Sgueglia G.A. (2012). A Randomized Multicenter Study Comparing a Paclitaxel Drug-Eluting Balloon with a Paclitaxel-Eluting Stent in Small Coronary Vessels: The BELLO (Balloon Elution and Late Loss Optimization) Study. J. Am. Coll. Cardiol..

[18] Yu X., Wang X., Ji F., Zhang W., Yang C., Xu F., Wang F. (2021). A Non-Inferiority, Randomized Clinical Trial Comparing Paclitaxel-Coated Balloon versus New-Generation Drug-Eluting Stents on Angiographic Outcomes for Coronary de Novo Lesions. Cardiovasc. Drugs Ther..

[19] Venetsanos D., Lawesson S.S., Panayi G., Tödt T., Berglund U., Swahn E., Alfredsson J. (2018). Long-Term Efficacy of Drug Coated Balloons Compared with New Generation Drug-Eluting Stents for the Treatment of de Novo Coronary Artery Lesions. Catheter. Cardiovasc. Interv..

[20] Gao C., He X., Ouyang F., Zhang Z., Shen G., Wu M., Yang P., Ma L., Yang F., Ji Z. (2024). Drug-Coated Balloon Angioplasty with Rescue Stenting versus Intended Stenting for the Treatment of Patients with de Novo Coronary Artery Lesions (REC-CAGEFREE I): An Open-Label, Randomised, Non-Inferiority Trial. Lancet.

[21] Zheng C., Kang J., Park K.W., Han J.-K., Yang H.-M., Kang H.-J., Koo B.-K., Kim H.-S. (2019). The Predictors of Target Lesion Revascularization and Rate of In-Stent Restenosis in the Second-Generation Drug-Eluting Stent Era. J. Interv. Cardiol..

[22] Sogabe K., Koide M., Fukui K., Kato Y., Kitajima H., Akabame S., Zen K., Nakamura T., Matoba S. (2020). Optical Coherence Tomography Analysis of Late Lumen Enlargement after Paclitaxel-Coated Balloon Angioplasty for De-Novo Coronary Artery Disease. Catheter. Cardiovasc. Interv..

[23] Her A.-Y., Ann S.H., Singh G.B., Kim Y.H., Okamura T., Garg S., Koo B.-K., Shin E.-S. (2016). Serial Morphological Changes of Side-Branch Ostium after Paclitaxel-Coated Balloon Treatment of de Novo Coronary Lesions of Main Vessels. Yonsei Med. J..

